# Ni(ii) immobilized on poly(guanidine–triazine–sulfonamide) (PGTSA/Ni): a mesoporous nanocatalyst for synthesis of imines†

**DOI:** 10.1039/d2ra06196a

**Published:** 2022-11-30

**Authors:** Ramin Ghiai, Sedigheh Alavinia, Ramin Ghorbani-Vaghei, Alireza Gharakhani

**Affiliations:** Department of Organic Chemistry, Faculty of Chemistry, Bu-Ali Sina University Hamedan 6517838683 Iran rgvaghei@yahoo.com ghorbani@basu.ac.ir +98 81 3838 0647

## Abstract

Mesoporous materials have been the subject of intense research regarding their unique structural and textural properties and successful applications in various fields. This study reports a novel approach for synthesizing a novel porous polymer stabilizer through condensation polymerization in which Fe_3_O_4_ magnetic nanoparticles (Fe_3_O_4_ MNPs) are used as hard templates. Using this method allowed the facile and fast removal of the template and mesopores formation following the Fe_3_O_4_ MNPs. Different techniques were performed to characterize the structure of the polymer. Based on the obtained results, the obtained mesoporous polymeric network was multi-layered and consisted of repeating units of sulfonamide, triazine, and guanidine as a novel heterogeneous multifunctional support. Afterward, the new nickel organometallic complex was supported on its inner surface using the porous poly sulfonamide triazine guanidine (PGTSA/Ni). In this process, the obtained PGTSA/Ni nanocomposite was used as a heterogeneous catalyst in the synthesis of imines from amines. Since this reaction has an acceptorless dehydrogenation pathway, the hydrogen gas is released as its by-product. The synthesized nanocatalyst was structurally confirmed using different characterization modalities, including FT-IR, SEM, XRD, EDX, TEM, elemental mapping, ICP-AES, BET, and TGA. In addition, all products were obtained in high turnover frequency (TOF) and turnover number (TON). The corresponding results revealed the high selectivity and activity of the prepared catalyst through these coupling reactions. Overall, the synthesized nanocatalyst is useable for eight cycles with no considerable catalytic efficiency loss.

## Abstract 2

Mesoporous materials have been the subject of intense research regarding their unique structural and textural properties and successful applications in various fields.

## Graphical abstract

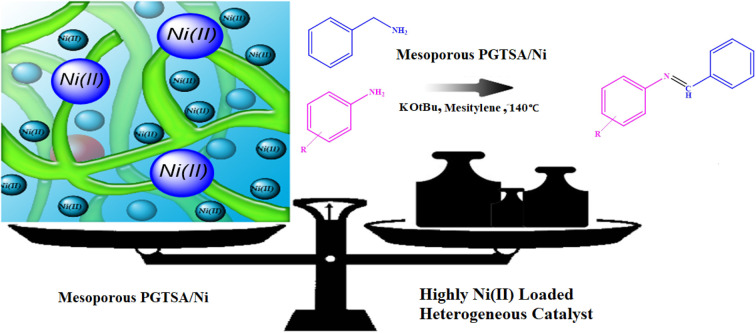

## 1. Introduction

Recently, acceptorless dehydrogenation of amines and alcohols has been considered among the strong tools for synthesizing different types of organic compounds.^1,2^ In these reactions, the primary alcohols and amines are converted to carbonyl species and imines as highly efficient intermediates for further transformations. This reaction releases hydrogen gas as its by-product. Unlike the common oxidation reactions, this reaction does not need a stoichiometric amount of a given additive or oxidant to take place.^3^

These transformations need expensive metals such as Ru and Ir, which have been successfully used for different types of organic reactions. Today, Earth-Abundant Metals (EAMs), such as Co, Mn, Fe, Zn, Mo, and Ni, are investigated through acceptorless dehydrogenative reactions as low-cost alternatives to replace costly rare metals.^3–5^ Unlike other EAMs, using Ni involves some benefits, including a wide range of ligands to use, low cost, and diversity of reactions to catalyze by Ni complexes. Nickel catalysts are widely used in carbon–heteroatom bonds in complex organic molecules, hydrogenation and reduction reactions,^6^ C–H bond activation reactions, multicomponent reaction^7^ and cross-coupling reactions to establish carbon–carbon bonds.^8–10^

In studying the dehydrogenation reactions through Ni-catalyzed acceptorless, Dai *et al.* prepared three Ni(ii) complexes with *N*′*NN*′ type pincer ligands to dehydrogenate primary alcohols to the carboxylic acids *via* releasing the hydrogen gas.^11^ Also, Parua *et al.* documented the mono- and double-dehydrogenative synthesis of quinolones derivatives. To this end, they used secondary alcohols through [Ni(tetramethyltetraaza[14]annulene)] and a complex of aminobenzyl alcohols with ketones as the active catalyst.^12^ Very recently, Liu *et al.* used RANEY®-Ni as a commercial Ni source to synthesize primary benzyl amines from ammonia and primary benzyl alcohols through a hydrogen borrowing pathway.^13^ In 2015, the preparation of nanoscale iron oxide-based materials and their use in the catalysis of different hydrogenation reaction was reported by Jagadeesh.^14^ Recently, cycloamination approaches for thermal (catalyst-free) and catalytic transformation of biomass feedstocks into N-heterocyclic molecules including mechanistic pathways are reported.^15^ In addition to cycloamination approaches, hydrothermal amination of wet biomass feedstocks is thus an attractive technical strategy for producing nitrogen-containing compounds.^16^ Regarding the critical role of catalysts in these reactions, researchers have constantly sought to develop recyclable, efficient, and environmentally friendly catalysts to meet the green chemistry requirements.

Nowadays, scientists endeavor to refine materials facilitating sustainable development.^17–20^ Mesoporous nanoarchitectures have been the subject of intense research regarding their extraordinary features.^21–23^ These nanostructures have found a wide range of applications in synthesizing catalysts,^24^ functional devices^25^ of these structures facilitates the reaction rate by permitting the passage of guest species to the available reaction points. These nanostructures include silica and non-siliceous materials (*e.g.*, carbon,^26^ metal oxides,^27^ inorganic–organic hybrid materials,^28^ polymers,^29^ and sulfides).^30^

Porous organic polymers (POPs) are another emerging material group that has received much attention. These polymers have several applications in pollutant removal,^31^ gas storage,^32^ photocatalyst,^33^ and catalyst.^34^ Mesoporous polysulfonamides, another growing branch of materials, have received much attention regarding their applications in heterogeneous catalysis and medicinal chemistry. Mesoporous polysulfonamides typically have low skeleton density, high surface area, deliberate tenability, and satisfactory chemical stability. A careful selection of monomers and templates plays an essential role in these materials' electronic, chemical, and topological properties.^6,34–37^ In general, nitrogenous ligands' incorporation into a polymer chain is an effective strategy to benefit simultaneously from the excellent characteristics of porous polymers and the common properties of ligands of nitrogen, thereby establishing other functionalities.

In this respect, researchers have successfully prepared a porous guanidine–triazine–sulfonamide-based polymer, as a new organic support system, *via* the Fe_3_O_4_ MNPs template technique. The repeated monomer units of guanidine, triazine, and sulfonamides in polymeric backbones form covalently active sites and offer high stability to immobilizing metal/metal NPs.

Most pure polymers have rare catalytic sites and small specific surface areas. The hard template is a convenient and effective strategy to prepare efficient pore structures for inorganic or inorganic–organic hybrid materials. Nevertheless, this method has rarely been used in pure organic polymers, as template removal is difficult. In this respect, hard template SiO_2_ particles have been used to prepare the pore structure of polysulfonamides. However, removing a template etched by NaOH solution is time-consuming, as the calcination process damages the framework. Hence, easily removable hard templates can be of particular importance in fabricating pore structures.^34^

**Scheme 1:**
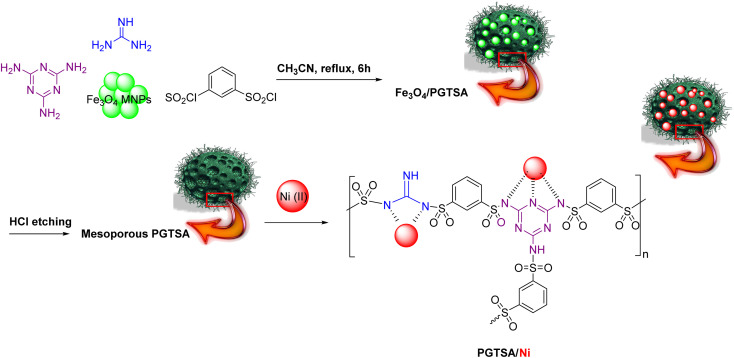
Schematic representation of synthesized PGTSA/Ni nanocomposite.

**Scheme 2:**
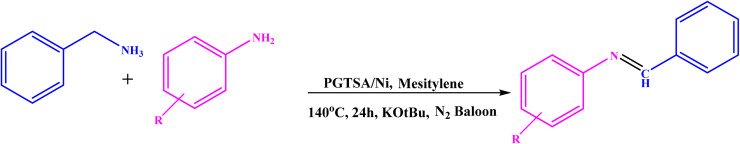
Benzylic amines dehydrogenation using PGTSA/Ni.

Following current efforts investigating environmentally friendly catalytic protocols for chemical synthesis^38–44^ in the present study, for the first time, we prepared a mesoporous PGTSA by applying condensation polymerization with a hard template of Fe_3_O_4_ particles. The template is easy to remove within a short time. In addition, the molecular structure of poly guanidine–triazole–sulfonamide effectively survived the template removal processes. Next, we functionalized with Ni(ii), characterized, and catalytically assessed the prepared poly guanidine–triazine–sulfonamide (Scheme 1). The results revealed that mesoporous PGTSA/Ni is useable as a suitable catalyst to couple amines with imines, regarding its properties such as multifunctional layers and strong metal sites (Scheme 2). Combining mesopore with N-containing ligands in a polymer matrix would significantly reduce the metal leaching in comparison to other mesoporous/microporous supports. Besides, this easily recoverable catalyst indicated high reusability and good features for green chemical production. Therefore, it can be regarded as a desirable stabilizer for synthesizing metallic nanocatalysts with high heterogeneity. The primary benefits of these supports are their facile preparation, need for chip raw materials, abundant precursors, versatility to composites, functionalizable surface, and chemical multifunctionality.

## 2. Experimental

### 2.1. General

All materials and reagents were purchased from chemical sources and used as received. The Fourier transform infrared spectroscopy (FT-IR) was performed using a Bruker Vertex 70 FT-IR spectrometer. ^1^H and ^13^C NMR spectra were recorded using a Bruker Avance 250 MHz spectrometer. The powder X-ray diffractometry (XRD) patterns were obtained using an X'Pert Pro Panalytical diffractometer applying a 30 mA current and a 40 kV voltage with Cu-Kα radiation (*λ* = 1.5418 Å). The morphology and size of the prepared nanoparticles were investigated using SEM images obtained from the FESEM-TESCAN MIRA3 instrument. Nanocatalysts' chemical composition was determined by EDX using SEM analysis. The specimen's magnetic susceptibility was determined *via* a vibrating sample magnetometer (Meghnatis Daghigh Kavir, Co. Iran) in a magnetic field of 20 kOe. Thermogravimetric analysis (TGA) was performed using the Shimadzu DTG-60 device at 0 to 700 °C. In addition, thin-layer chromatography (TLC) analysis was performed to determine the products' purity and monitor the reaction progress in a silica gel 60 F254 aluminum sheet.

### 2.2. Fe_3_O_4_ MNPs synthesis

Fe_3_O_4_ magnetic nanoparticles (Fe_3_O_4_ MNPs) were synthesized through the co-precipitation approach described in the literature.^39^ Briefly, 3 mL of FeCl_3_ (2 M dissolved in 2 M HCl) was added to 10.33 mL of double distilled water. Next, Na_2_SO_3_ (2 mL, 1 M) was added to the solution dropwise for 3 min under magnetic stirring. Once the solution color turned yellow from red, 80 mL of 0.85 M NH_3_·H_2_O solution was added to it while vigorously stirring. After 30 min, an external magnet was used to separate the prepared Fe_3_O_4_ MNPs. Eventually, they were washed with distilled water to reach a pH of less than 7.5.

### 2.3. Fe_3_O_4_/PGTSA nanocomposite synthesis

Fe_3_O_4_/PGTSA nanocomposite was synthesized through *in situ* polymerization of guanidine (0.3 mol), 1,3,5-triazine-2,4,6-triamine (0.7 mol), and benzene-1,3-disulfonyl chloride (1 mol) in the presence of acetonitrile (10 mL) and Fe_3_O_4_ MNPs (0.1 g). Next, the reaction mixture was stirred for 6 h under reflux conditions. The nanocomposites were filtered out and washed with water and ethanol. The prepared solid residue was dried under vacuum conditions at room temperature.

### 2.4. Porous PGTSA synthesis

Fe_3_O_4_ MNPs were etched using an HCl aqueous solution to selectively remove the Fe_3_O_4_ MNPs of the Fe_3_O_4_/PGTSA nanocomposite. Next, Fe_3_O_4_/PGTSA nanocomposite (0.5 g), HCl solution (10 mL, 10 wt%), deionized water (10 mL) were added to a 5 mL flask. Afterward, the reaction mixture was shaken for 15 min at room temperature. Finally, the prepared porous polymer was washed with water and dried at 50 °C.

### 2.5. Porous PEMA-PSA/Ni preparation

We sonicated the synthesized polymeric support (0.1 g) in an aqueous solution of NiCl_2_·6H_2_O (2 mL, 1 M) for 15 min, followed by its stirring for 5 h at room temperature. The synthesized catalyst was filtered out and washed using excess water and ethanol. In the end, the prepared residue was dried at room temperature under vacuum conditions.

### 2.6. Dehydrogenative of imines synthesis

A solution of amine (2 mmol), benzylamines (1 mmol), potassium *tert*butoxide (0.25 mmol) and PGTSA/Ni (0.9 mol%) was stirred in mesitylene (4 mL) at 140 °C under N_2_ atmosphere. After completion of the reaction (monitored by TLC), PGTSA/Ni catalyst was isolated by an centrifugation, washed with EtOH and EtOAc (2 × 5 mL) and dried under vacuum. The title compounds were obtained in their crystalline forms by recrystallization of ethanol solution.

## 3. Results and discussion

### 3.1. Characterization of PGTSA/Ni nanocatalyst

**Fig. 1:**
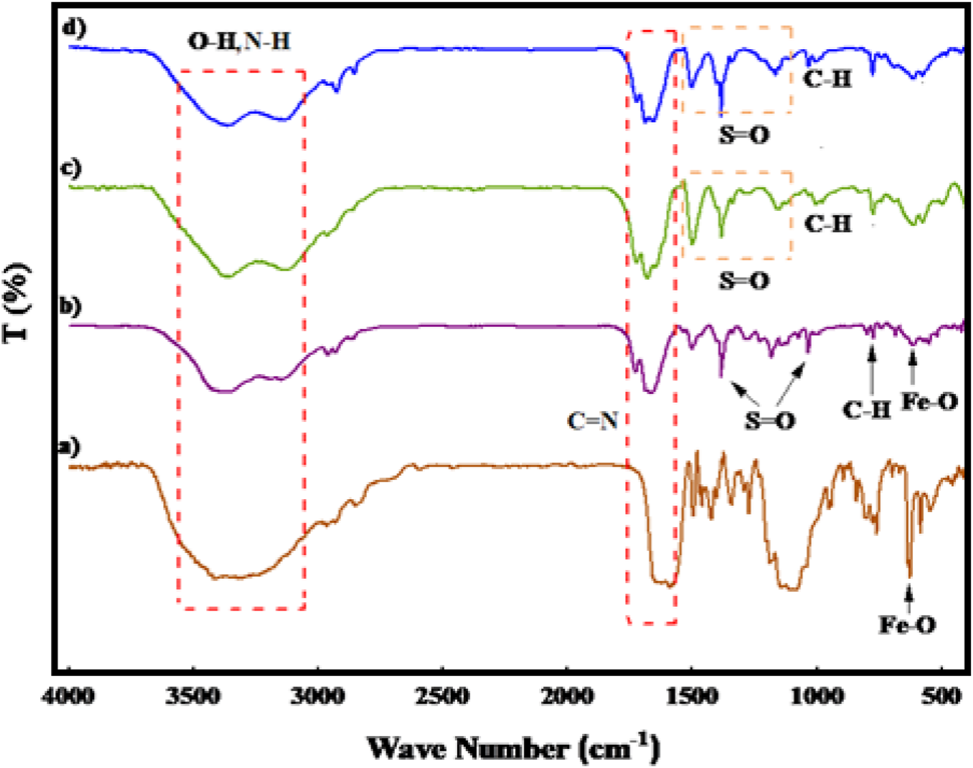
FT-IR spectra of Fe_3_O_4_ MNPs (a), Fe_3_O_4_/PGTSA (b), mesoporous PGTSA (c), and PGTSA/Ni (d).

**Fig. 2:**
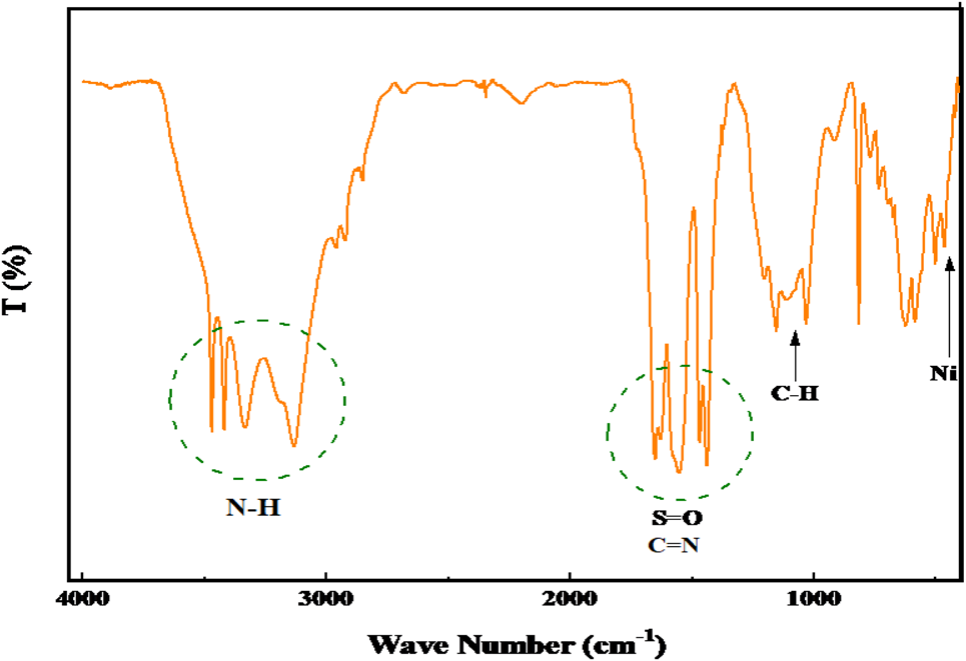
FT-IR spectra of recycled PGTSA/Ni after seven cycle.

Fig. 1 illustrates the FT-IR spectra of PGTSA/Ni, Fe_3_O_4_ MNPs, mesoporous PGTSA, and Fe_3_O_4_/PGTSA. In Fig. 1a, the Fe–O stretching vibration of Fe_3_O_4_ appeared at about 627 cm^−1^. In the FT-IR spectra of Fe_3_O_4_/PGTSA, Fe_3_O_4_ MNPs are confirmed by the peaks at 605 cm^−1^. This peak is indexed to magnetite's Fe–O stretching oscillation. Tensile vibrations related to C=N and S=O appeared at 1644, 1660 cm^−1^, and 1032, 1384 cm^−1^, respectively (Fig. 1b). The spectrum of mesoporous PGTSA revealed characteristic peaks similar to those of Fe_3_O_4_/PGTSA, but not any characteristic peaks for Fe_3_O_4_. Therefore, it is inferred that the PGTSA structure survived the polymerization and template removal processes (Fig. 1c). In the PGTSA/Ni catalyst case, the C=N/S=O vibration moved to a lower wavenumber. The observed shift peaks indicate the successful coordination of Ni within the polymer ligands (Fig. 1d). Fig. 2 presents the catalyst FTIR spectrum after seven reuse cycles. The figure indicates no significant alteration in the reused catalyst's FTIR spectrum, suggesting the negligible effect of the catalytic reaction on the catalyst's chemical structure.

**Fig. 3:**
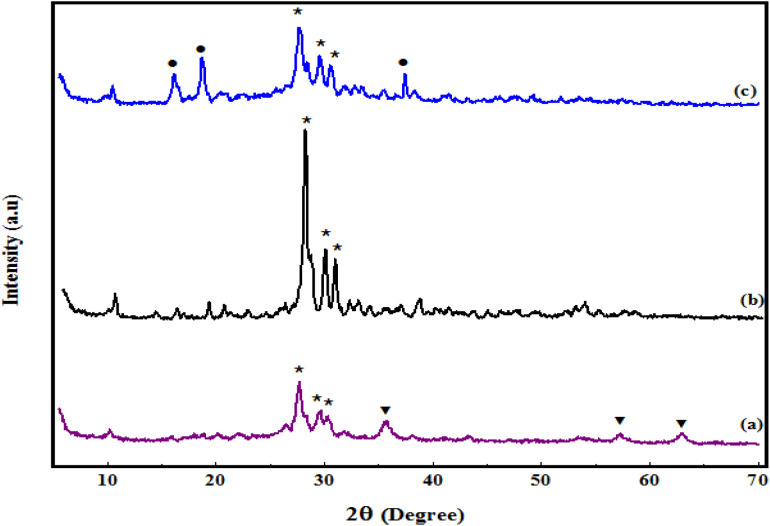
XRD pattern of Fe_3_O_4_/PGTSA (a), porous PGTSA (b), and PGTSA/Ni (c).

The normal XRD patterns of Fe_3_O_4_/PGTSA, mesoporous PGTSA, and mesoporous PGTSA/Ni are shown in Fig. 3. The Fe_3_O_4_/PGTSA pattern includes two groups of diffraction peaks: (1) the diffraction peaks of PGTSA and (2) the diffraction peaks of Fe_3_O_4_. These peaks indicate the polymeric hybrid's crystalline structure. The Fe_3_O_4_/PGTSA's normal XRD pattern revealed three peaks at 2*θ* = 35.47°, 57.42°, and 62.87° indexed to Fe_3_O_4_ MNPs' crystal phase.^38^ Moreover, the peak at 2*θ* = 27.8°, 29.72°, and 30.67° are attributed to the presence of guanidine, triazine, and sulfonamides of PGTSA (Fig. 3a). The mesoporous PGTSA had the diffraction peaks of guanidine, triazine, and sulfonamides, but without any Fe_3_O_4_ diffraction peak. This outcome indicates the crystalline structure of the polymer and the complete total of the Fe_3_O_4_ template. Furthermore, these results confirmed that the template removal did not alter the crystal phase of Fe_3_O_4_/PGTSA (Fig. 3b). Based on the final catalyst's XRD pattern, adding NiCl_2_ to mesoporous PGTSA has altered the mesoporous PGTSA's crystalline structure, due to the interactions between Ni(ii) and the prepared support. Besides, the peaks at 2*θ* = 16.02°, 18.67°, and 37.47° are indexed to nickel groups.^6^ This result confirmed the successful Ni immobilization on mesoporous PGTSA (Fig. 3c).

**Fig. 4:**
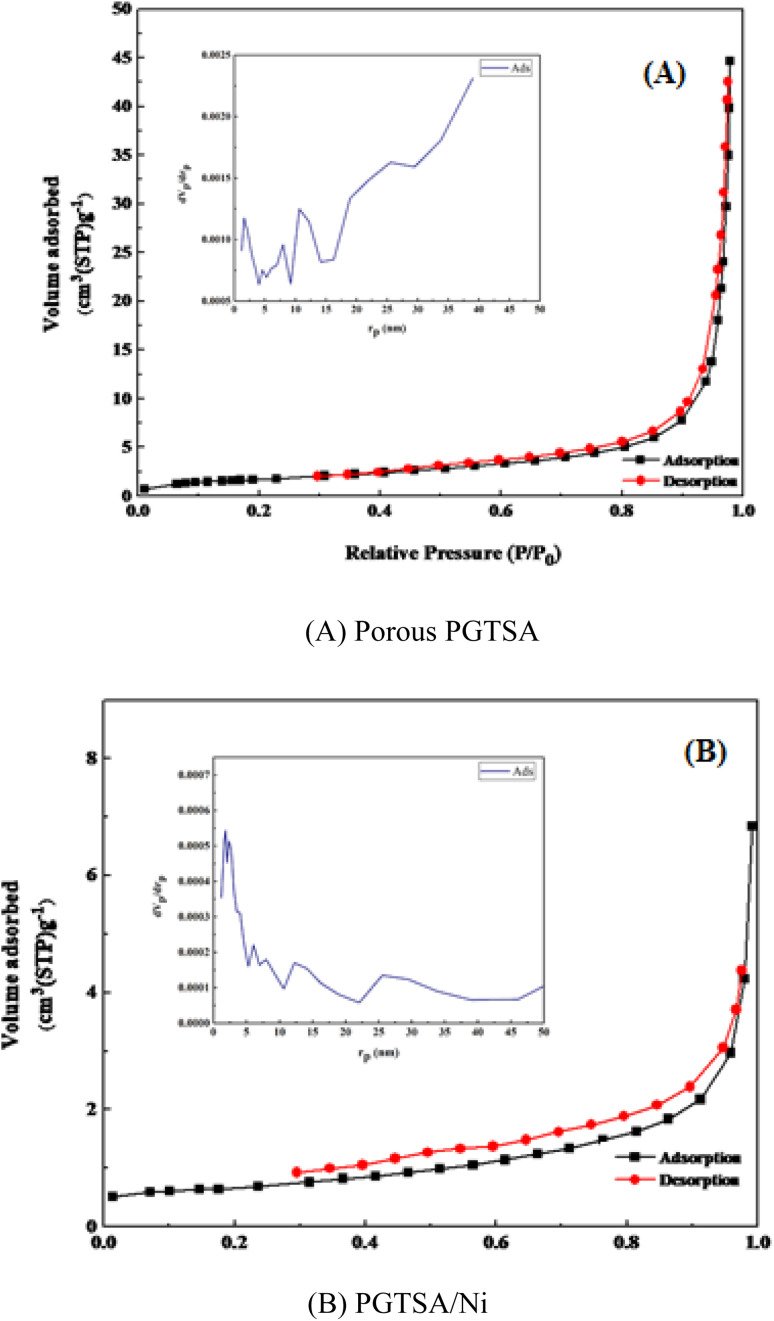
N_2_ adsorption–desorption isotherms of (A) porous PGTSA, and PGTSA/Ni (B).

Brunauer–Emmett–Teller (BET) analysis was performed to prepare the N_2_ adsorption/desorption isotherms of the prepared PGTSA nanostructures. This experiment gives information about the specific surface area (SSA) and porosity of the prepared nanostructural PGTSA. We observed a distinctive H4-type hysteresis loop of type IV for the PGTSA and PGTSA/Ni, indicating the development of ordered mesoporous structures. These isotherms show the BET SSA of 11.81 m^2^ g^−1^, a pore mean diameter of 39.7 nm, and a pore volume of 0.058 cm^3^ g^−1^ for mesoporous PGTSA (Fig. 4a). As can be inferred from N_2_ adsorption/desorption isotherms of PGTSA/Ni, the SSA, pore volume, and pore mean diameter of this structure are 3.6556 m^2^ g^−1^, 0.0098 cm^3^ g^−1^, and 1.86 nm, respectively (Fig. 4b). It is noteworthy that SSA of PGTSA/Ni is lower than PGTSA. This difference might be due to the grafting of Ni-complex on mesoporous channels of PGTSA (Table 1).

**Table 1:** Results of the Langmuir and BET measurements of mesoporous PGTSA and PGTSA/Ni

Parameter	PGTSA	PGTSA/Ni
*a*_s_ (m^2^ g^−1^)	11.81	3.6556
*V*_m_ (cm^3^(STP) g^−1^)	2.71	0.5418
*V*_p_ (cm^3^ g^−1^)	0.058	0.0098
*r*_p_ (nm)	39.7	1.86
*a*_p_ (m^2^ g^−1^)	7.33	1.685

**Fig. 5:**
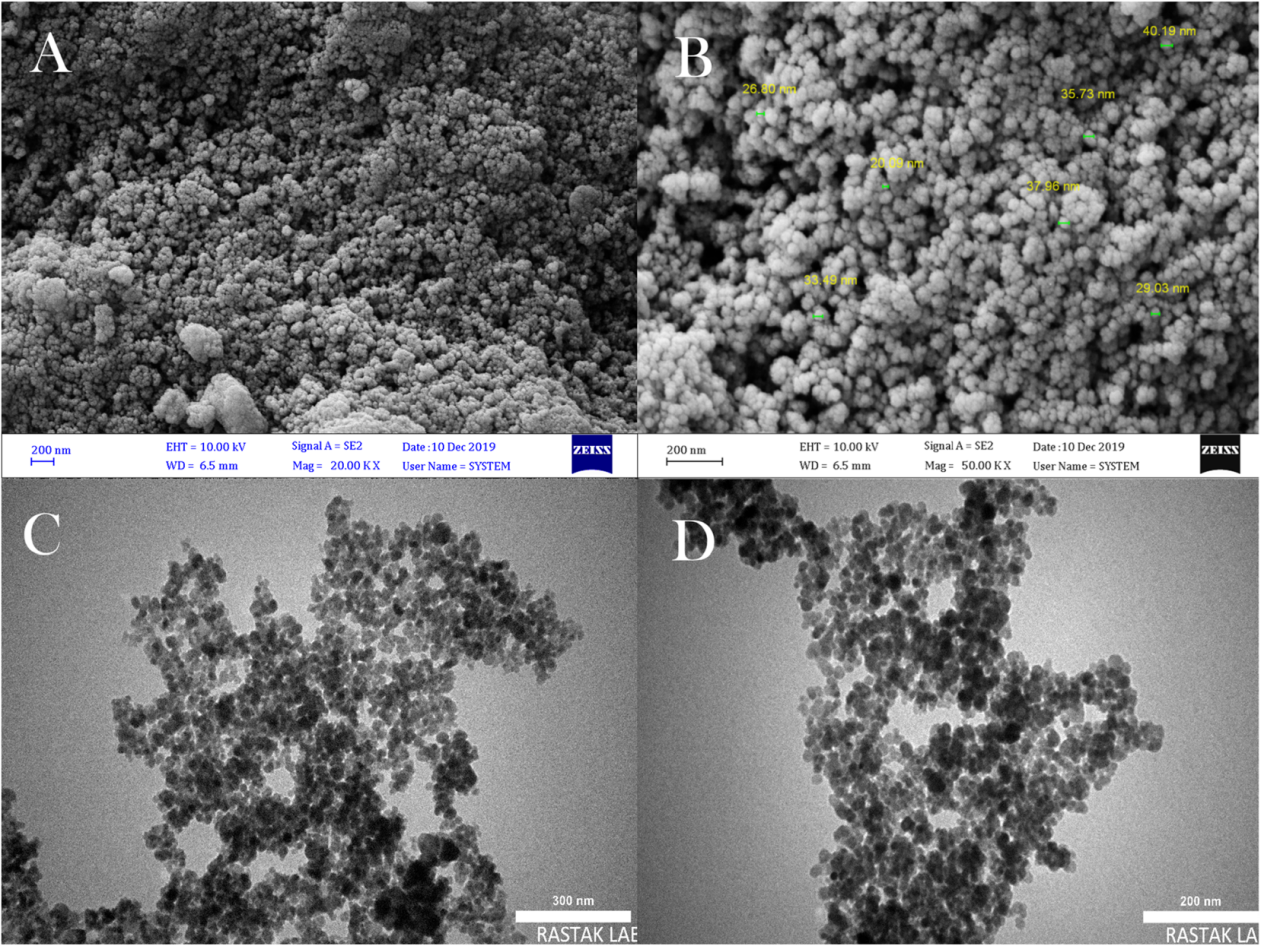
FESEM images of Fe_3_O_4_ MNPs (A and B), TEM images of Fe_3_O_4_ MNPs (C and D).

Fig. 5 shows the prepared FE-SEM and TEM to characterize the shape, size, and morphology of the synthesized Fe_3_O_4_ MNPs template. As can be seen from the SEM image of NMPs, these nanoparticles are monodispersed and spherical, have an almost narrow size distribution, and have a mean diameter of 20–50 nm (Fig. 5A and B). Also, like the SEM images, TEM images verified the spherical shape of Fe_3_O_4_ MNPs (Fig. 5C and D).

**Fig. 6:**
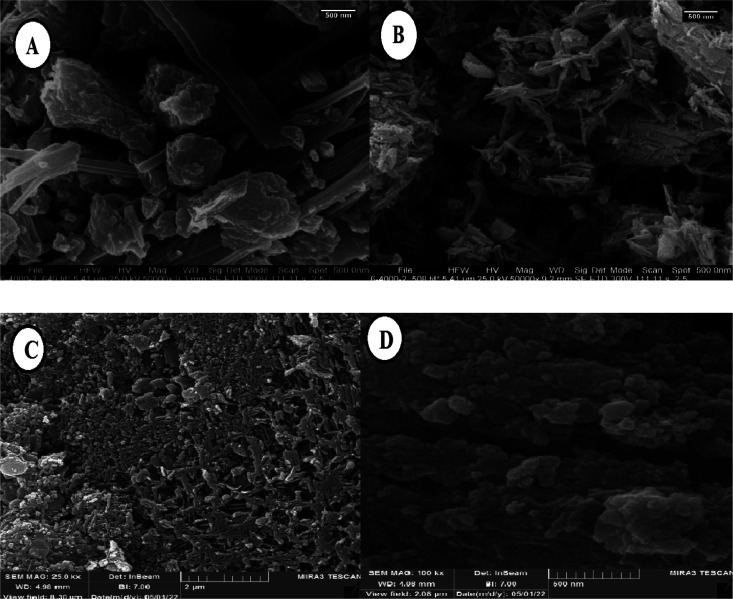
FESEM images of Fe_3_O_4_/PGTSA MNPs (A), PGTSA (B), porous PGTSA (C), PGTSA/Ni (D).

The shape and surface morphology of Fe_3_O_4_/PGTSA MNPs, mesoporous PGTSA, and PGTSA/Ni were investigated by FESEM (Fig. 6). FESEM image of Fe_3_O_4_/PGTSA MNPs revealed the spherical morphology of Fe_3_O_4_ MNPs and layered composite-type structure of PGTSA (Fig. 6A). FE-SEM images of PGTSA (Fig. 6B) showed a large block-like morphology without any porous structures. FE-SEM images of mesoporous PGTSA were captured for Fe_3_O_4_/PGTSA nanocomposite through selective removal of Fe_3_O_4_ MNPs template. These images show a uniform 3D porous network with a large number of multiple spherical-nano pores. In comparison, the template-free PGTSA synthesized under similar conditions showed a uniform structure of PGTSA regarding its robust nature, good distribution, and porosity. The mesoporous PGTSA showed a monolith morphology with abundant mesopores and a porous structure. These characteristics show the survival of the PGTSA's molecular structure from polymerization and template removal processes (Fig. 6C). The interactions between support and NiCl_2_ caused the formation of a porous aggregated 3-D framework (Fig. 6D). Such a network increased the catalytic properties of the synthesized nanostructured PGTSA/Ni.

**Fig. 7:**
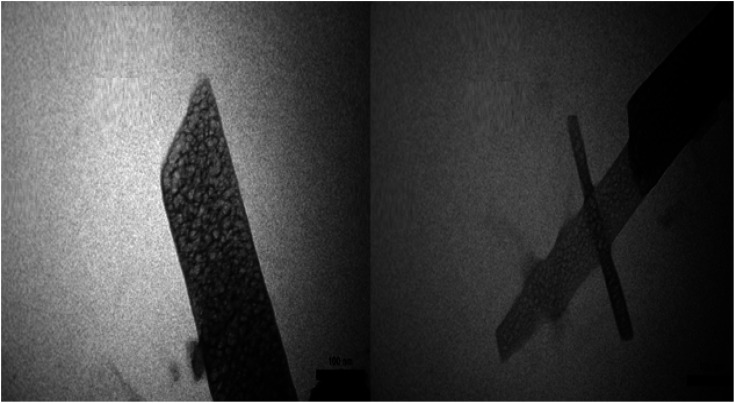
TEM images of porous PGTSA with the scale bar of 100 nm.

The captured TEM micrographs were analyzed to study the particle distribution, size, and morphology of mesoporous PGTSA. TEM images of PGTSA show the mesoporous structure with no aggregation (Fig. 7).

**Fig. 8:**
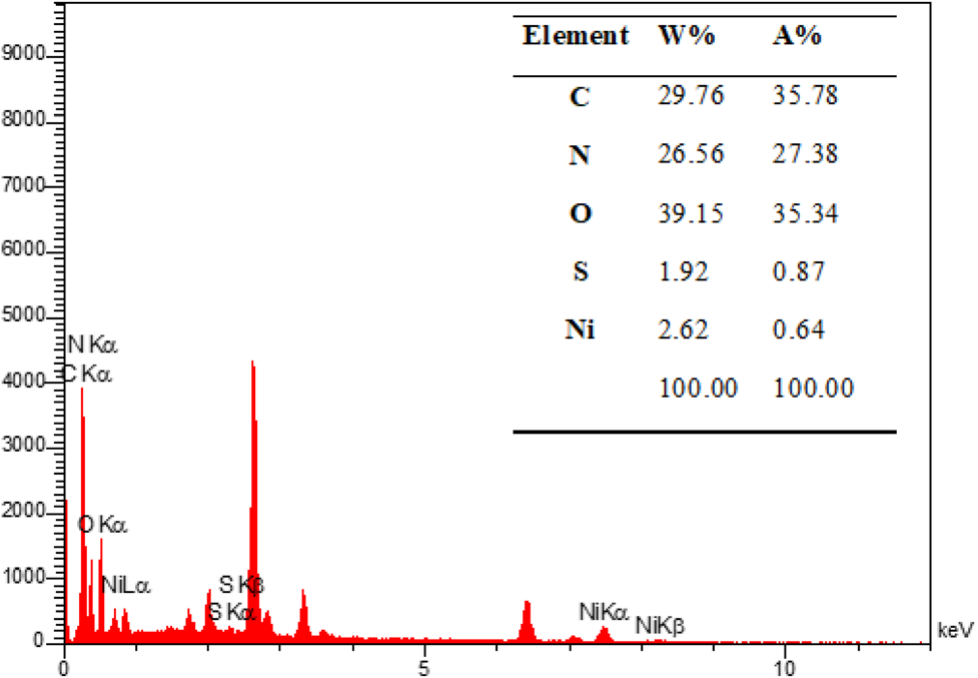
EDX spectrum of PGTSA/Ni.

**Fig. 9:**
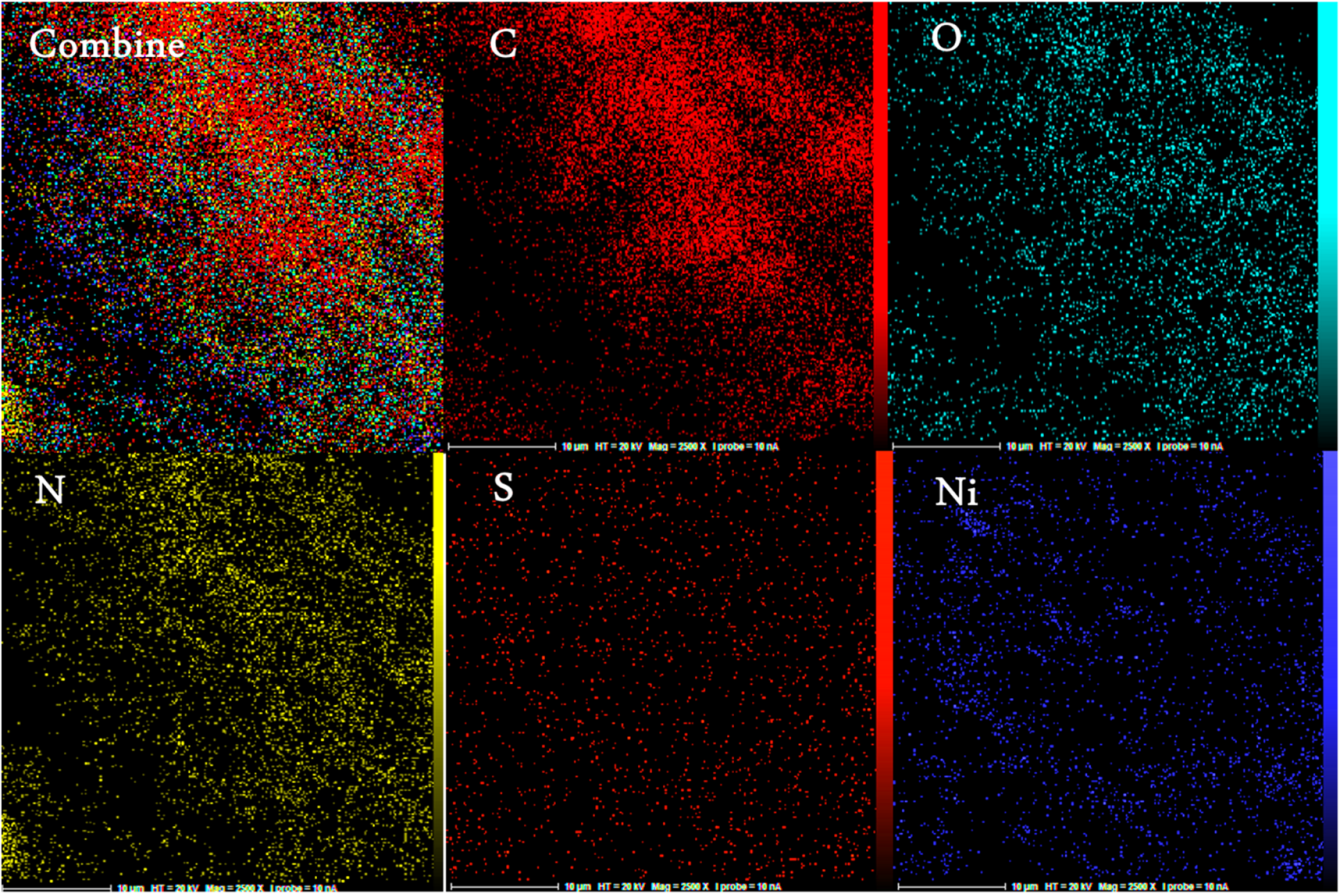
Elemental mapping of the C, N, O, S, and S atoms achieved from SEM micrographs.

The energy-dispersive X-ray spectroscopy (EDX) was performed to confirm the mesoporous PGTSA/Ni's elemental composition (Fig. 8). The results showed the presence of elements such as C, N, O, Ni, and S in the prepared catalyst. Fig. 9 shows the uniform distribution of C, S, O, and N and the perfect uniform distribution of Ni in this composite structure.

**Fig. 10:**
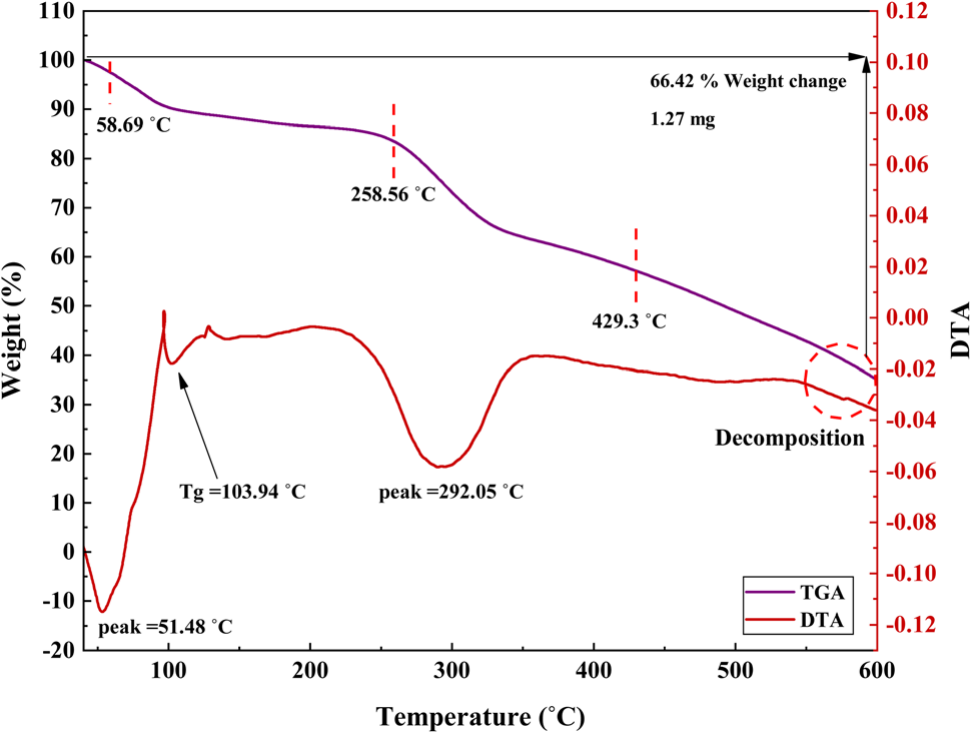
TGA curve of PGTSA/Ni.

Weight loss and stability of the synthesized catalyst were investigated using the thermogravimetric analysis (TGA). As observed from the curve in Fig. 10, losing 2.7% of the weight between 40–150 °C could be related to loss of moisture and solvents. Also, weight loss at 150–600 °C indicates the removal of organic moieties such as triazine, 1,3-benzene-disulfonyl chloride, and decomposition of the composite. The TGA curve of PGTSA/Ni shows a five-step degradation, by which 85.54% of the sample was lost at 100, 129, 250.6, 330, and 384 °C, respectively. In addition, according to the DTA diagrams, the glass transition point (*T*_g_) was 129 °C.

### 3.2. Catalytic activity studies of mesoporous PGTSA/Ni

After its full characterization, PGTSA/Ni's catalytic activity was investigated for the aniline and benzylamine reaction to optimize the reaction conditions (Table 2). In this study, the formation of a homo-coupling adduct was prevented by using two equivalents of aniline for an equivalent of benzylamine. Next, the corresponding impacts of the base, catalyst, and solvent amounts and temperature were explored in the model reaction. First, the catalytic activity of NiCl_2_ and CoCl_2_ were studied to compare the performance of these catalysts, which showed an unsuccessful reaction. Next, the model reaction was investigated in the presence of PGTSA/Ni under base-free conditions. The imine adduct was gained in a 61% isolated yield. Next, the reaction models were carried out using different amounts of catalyst in the presence of KO*t*Bu. The highest yield within the appropriate time was obtained when using 0.9 mol% of catalyst (entry 3). Exceeding the catalyst amount by 0.9 mol% did not affect the reaction yield (entries 4–6). Afterward, the model reaction was carried out using different amounts of bases, such as KOH and K_2_CO_3_. Based on the obtained results, the best yield (*i.e.*, 89%) was obtained when using KO*t*Bu in the model reaction (entries 7 and 8). In the next step, the best solvent in the reaction was optimized by screening the probe in several solvents, including CH_3_CN, EtOH, mesitylene, and toluene, and under solvent-free conditions (entries 9–13). The results showed that the polar and protic solvents declined the reaction rate, and mesitylene provided an excellent yield. Evaluating the effect of the temperature on the reaction model revealed that at low temperatures, the reaction did not progress significantly, and the maximum yield was gained by carrying out the reaction at 140 °C (entries 14 and 15). Eventually, adding 1 mmol of benzylamine to the reaction with 1 equivalent of amine, the corresponding imine was generated with a yield of 58% (entry 16). Screening the results showed that the optimal yield is achieved when 0.9 mol% PGTSA/Ni catalyst is added to mesitylene as the solvent at 140 °C (entry 5). Besides, to show the practical use of the PGTSA/Ni nanostructure, an experiment on a gram scale was carried out through the model reaction, and the desired yield was achieved (entry 17). The excellent catalytic performance of the PGTSA/Ni in the aprotic solvents can be attributed to PGTSA/Ni's amphiphilic behavior using hydrophobic monomers (aromatic groups) and hydrophilic monomers (*i.e.*, Ni, guanidine, and triazine). Further, PGTSA/Ni activates the intermediates *via* H-bonding with PGTSA/Ni's polar functional groups.

**Table 2:** PGTSA/Ni-catalyzed dehydrogenative heterocoupling of amines^a^

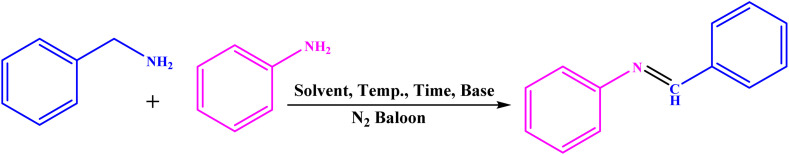							
Entry	Catalyst	Cat. (mol%)	Solvent	Temperature (°C)	Time (h)	Base	Yield^b^ (%)
1	NiCl_2_	50 mg	Mesitylene	140	24	KO*t*Bu	40
2	CoCl_2_	50 mg	Mesitylene	140	24	KO*t*Bu	N.R.
3	PGTSA/Ni	0.9 mol%	Mesitylene	140	24	—	60
4	PGTSA/Ni	0.45 mol%	Mesitylene	140	24	KO*t*Bu	72
5	PGTSA/Ni	0.9 mol%	Mesitylene	140	24	KO*t*Bu	93
6	PGTSA/Ni	1.8 mol%	Mesitylene	140	24	KO*t*Bu	93
7	PGTSA/Ni	0.9 mol%	Mesitylene	140	24	KOH	75
8	PGTSA/Ni	0.9 mol%	Mesitylene	140	24	K_2_CO_3_	88
9	PGTSA/Ni	0.9 mol%	Mesitylene	140	24	KO*t*Bu	69
10	PGTSA/Ni	0.9 mol%	Toluene	Reflux	24	KO*t*Bu	73
11	PGTSA/Ni	0.9 mol%	EtOH	Reflux	24	KO*t*Bu	40
12	PGTSA/Ni	0.9 mol%	CH_3_CN	Reflux	24	KO*t*Bu	52
13	PGTSA/Ni	0.9 mol%	Solvent-free	140	24	KO*t*Bu	32
14	PGTSA/Ni	0.9 mol%	Mesitylene	160	24	KO*t*Bu	93
15	PGTSA/Ni	0.9 mol%	Mesitylene	120	24	KO*t*Bu	64
16	PGTSA/Ni	0.9 mol%	Mesitylene	140	24	KO*t*Bu	58^c^
17	PGTSA/Ni	0.9 mol%	Mesitylene	140	24	KO*t*Bu	89^d^

a Reaction conditions: 1a (1.0 mmol), 2a (2.0 mmol), and 3 mL of solvent.

b Isolated yield.

c 1 mmol of aniline was employed.

d The reaction was carried out in 10 mmol scale.

Under optimized reaction conditions, reaction scope was explored using various aromatic amines. First, some aromatic amines chosen for the desired imines were prepared with a yield of 78–93% (Table 3; entries 3, 8, and 10). As can be seen from Table 3, ERGs existing in aniline derivatives can intensify the reactants' reactivity. On the other hand, the EWG existing on the phenyl ring can lower the reaction yield.

**Table 3:** Synthesis of imines from different amines using PGTSA/Ni^a^

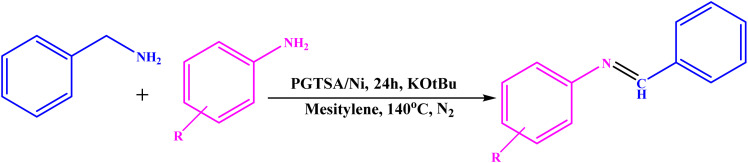					
Entry	Substrate	Product	Yield (%)	Melting point	
				Measured	Literature
1	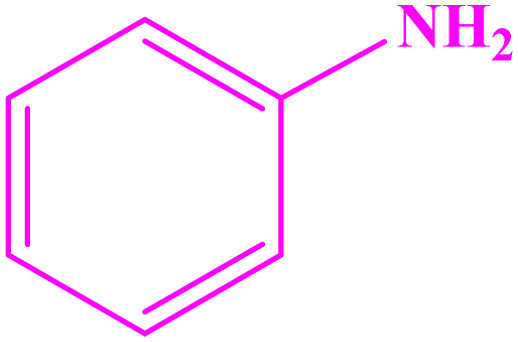	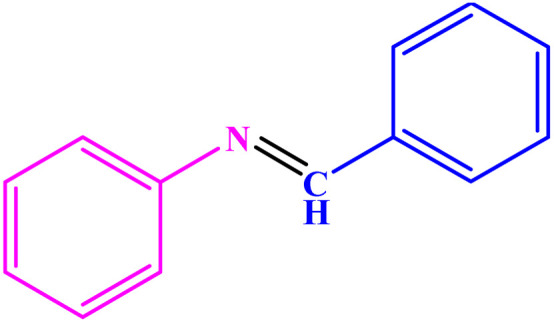	93	50–52	51–52 (ref. 45)
2	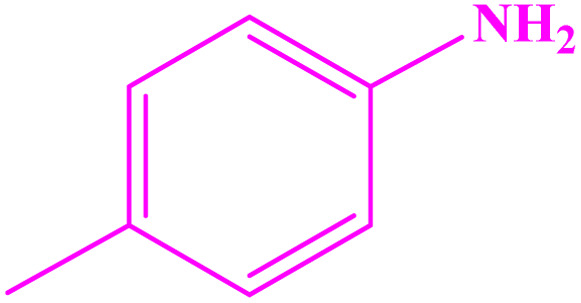	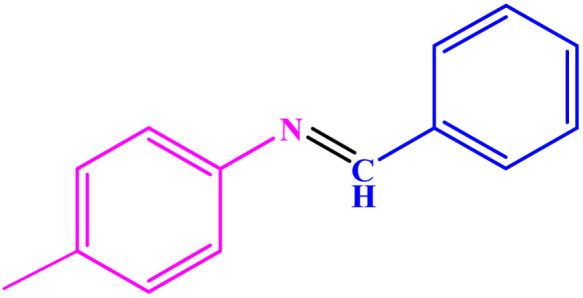	95	82–83	88–90 (ref. 46)
3	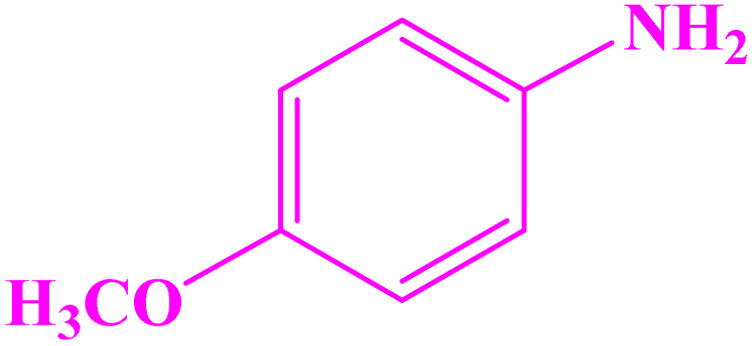	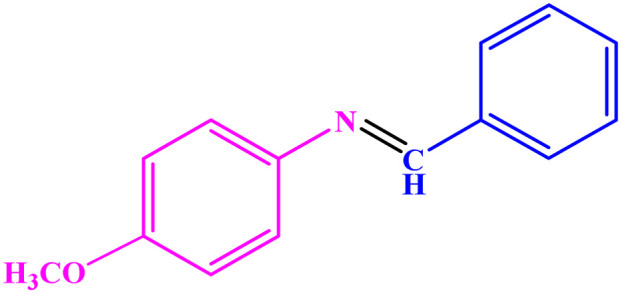	98	68–70	68–70 (ref. 47)
4	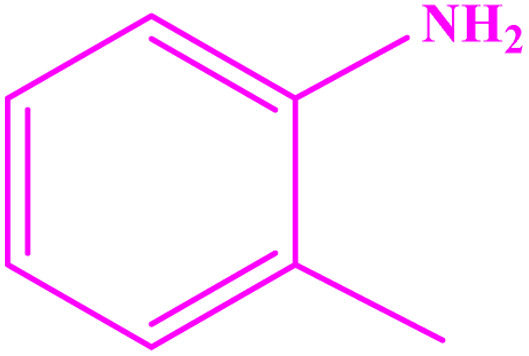	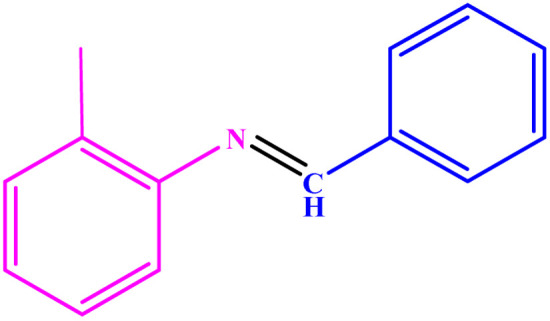	90	43–45	40 (ref. 48)
5	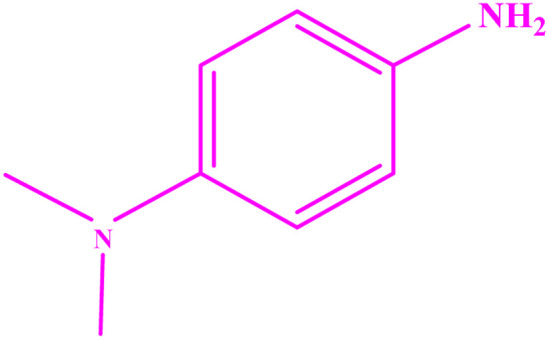	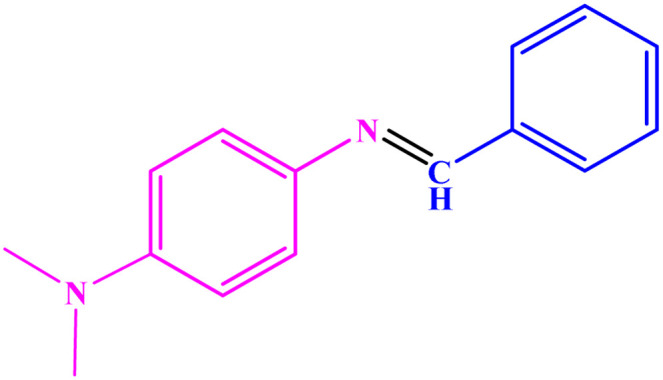	93	97–98	95–96 (ref. 49)
6	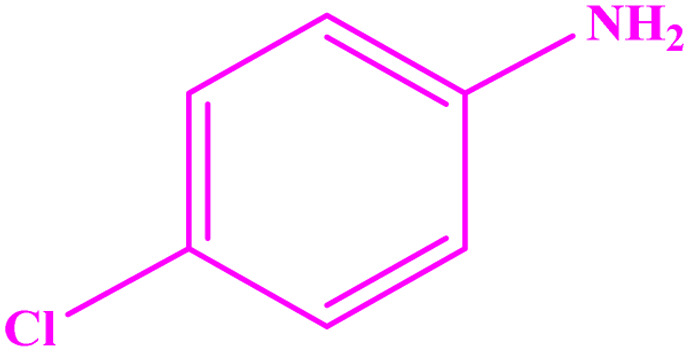	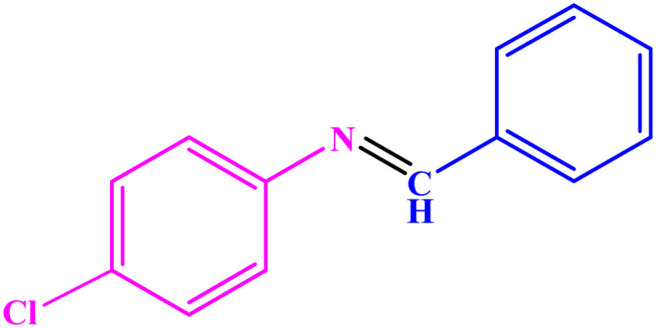	85	58–60	58–60 (ref. 50)
7	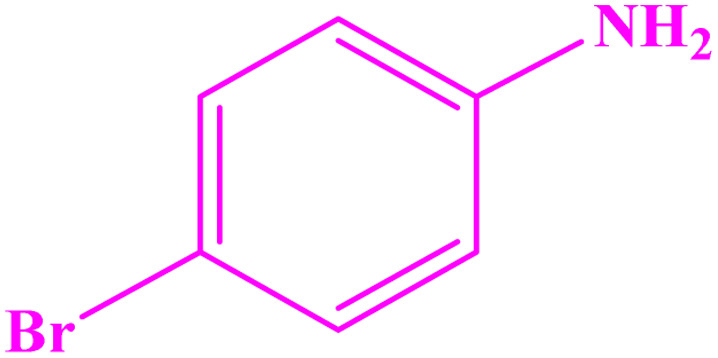	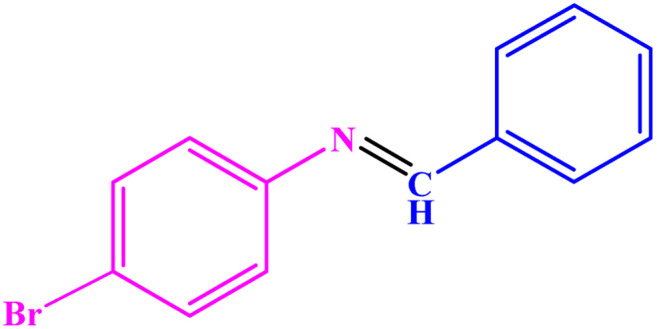	88	61–62	64–66 (ref. 51)
8	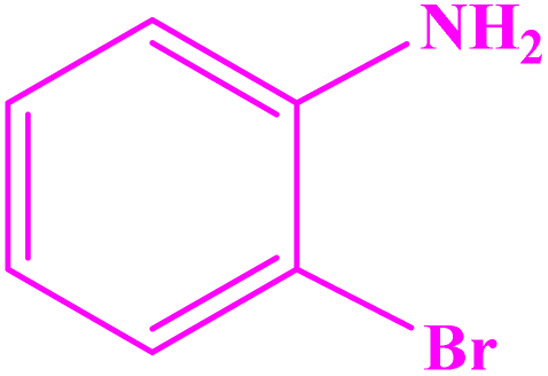	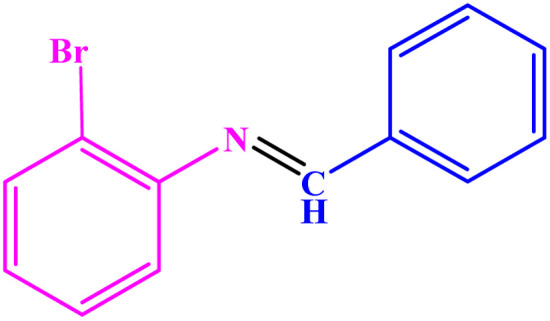	83	45	43–44 (ref. 52)
9	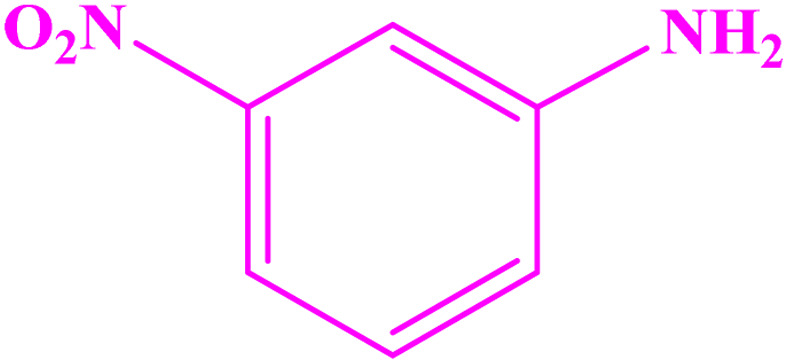	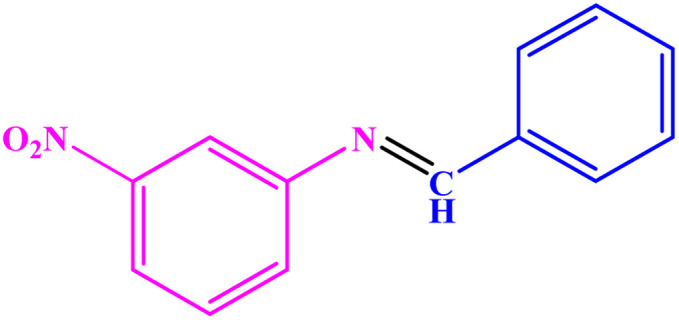	80	69–70	68–70 (ref. 53)
10	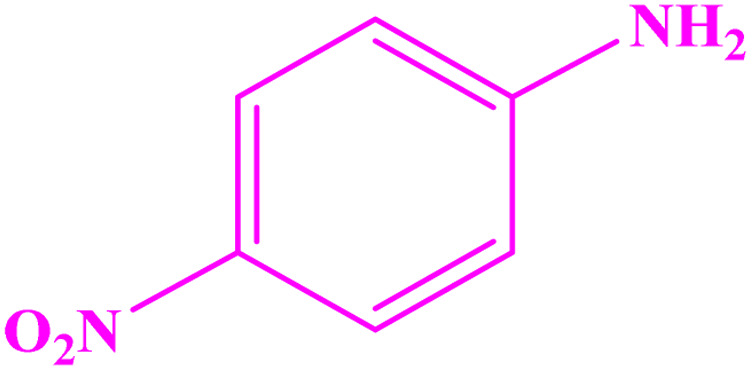	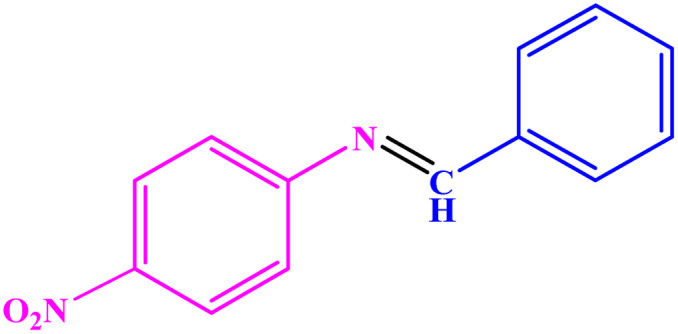	77	120–122	121–123 (ref. 53)

a Reaction condition: a solution of amine (2 mmol), benzylamines (1 mmol), potassium *tert*butoxide (0.25 mmol) and PGTSA/Ni (0.9 mol%) was stirred in mesitylene (4 mL) at 140 °C under N_2_ atmosphere.

**Scheme 3:**
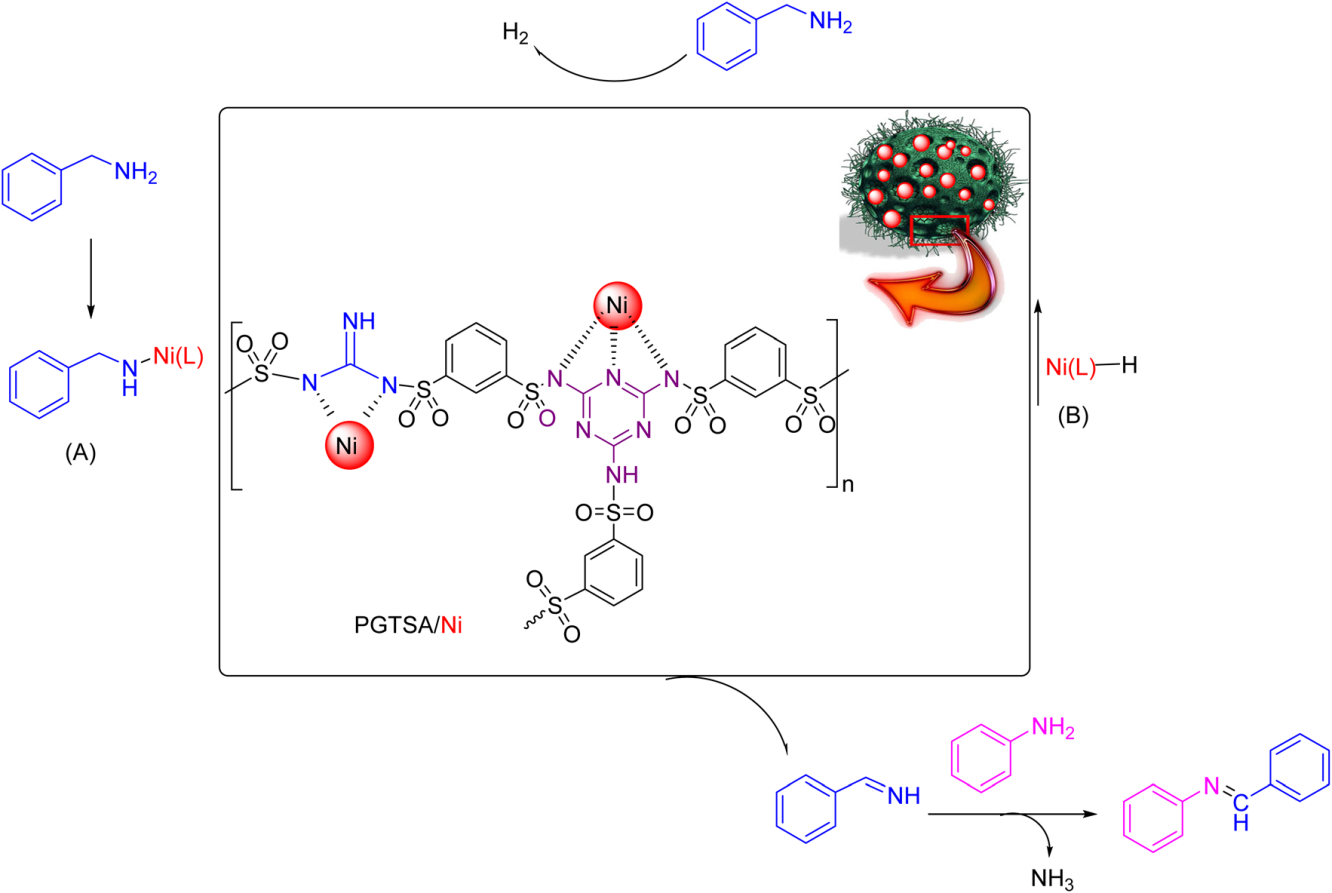
A plausible mechanism for the preparation of imines using mesoporous PGTSA/Ni.

Dangling hydrophobic and hydrophilic moieties on the PGTSA/Ni play a key role in the reactant diffusion into the mesitylene *via* establishing H-bonding between the dangling polar groups of aniline/benzylamine and the catalyst. Next, the ligand exchange occurs between PGTSA/Ni and aniline/benzylamine. As a result, the intended imine compound is generated in high yield by benzylamine dehydration. Scheme 2 presents a feasible mechanism for this catalytic system in the acceptorless dehydrogenation of benzylamine and its derivatives. Initially, intermediate A is formed by the coordination of benzylamine to Ni(L) in the presence of a base. Next, in line with β-H elimination of A, an aldimine intermediate and an Ni-hydride intermediate B are formed. The obtained aldimine forms the corresponding imine by subjecting it to a condensation reaction with RNH_2_ (Scheme 3).^54^

According to the ICP analysis and catalytic performance, mesoporous PGTSA/Ni had a more intense catalytic activity than Fe_3_O_4_/PGTSA/Ni and PGTSA/Ni (Table 3). In this research, the Ni contents of Fe_3_O_4_/PGTSA/Ni and PGTSA/Ni were 3.41% and 2.61%, respectively, which were lower than that of mesoporous PGTSA/Ni (5.28%). This difference might be due to high surface area and the cooperative interaction of triazine, sulfonamide, and guanidine groups with nickel. These functional groups can be used to stabilize the Ni *via* a much stronger bonding interaction between mesoporous polymer and Ni compared to that between Fe_3_O_4_/PGTSA and PGTSA. The results indicate the combination of high surface area and functional groups play essential role in activation of Ni *via* stabilization of Ni and diffusion of substrates *via* π–π interaction and hydrogen bonding.

**Scheme 4:**
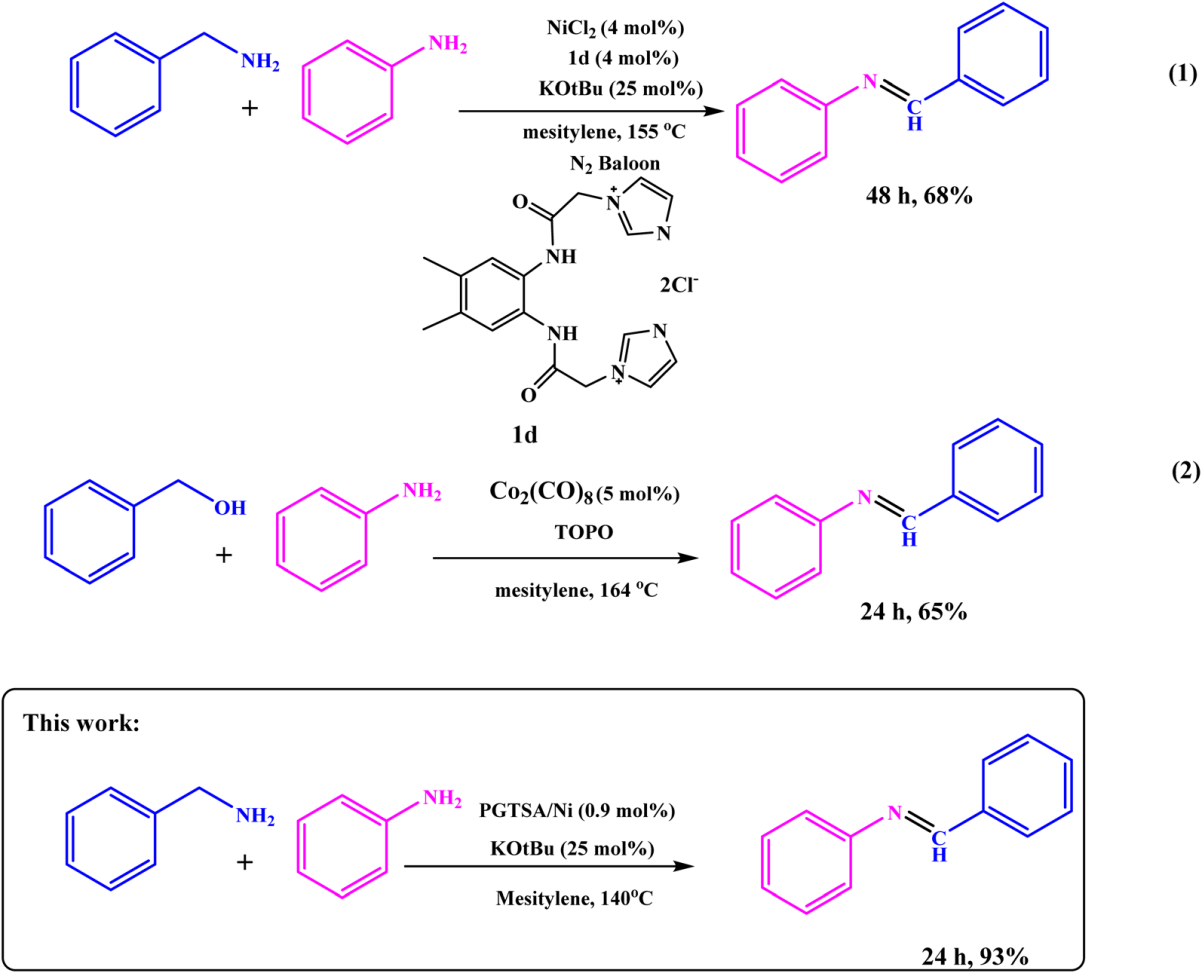
Literature precedent to synthesis of imines.

Next, to show the merits of the present method for the synthesis of imines from benzylamine and aniline, the catalyst efficiency of the mesoporous PGTSA/Ni was compared with the other catalysts which are reported in the literature (Table 4). The formation of title compounds was reported under the following conditions that contains longer reaction times and hazardous metal catalyst under drastic heating conditions. As shown in Scheme 4, Takallou and co-workers reported the Ni-catalyzed the preparation of imines from amines (eqn (1)).^54^ In 2019, Co-catalyzed dehydrogenative coupling of amines into imines (eqn (2)).^55^ This comparison shows that using mesoporous PGTSA/Ni system lead to synthesis products in higher yields times. The catalyst is composed of triazine, sulfonamide, and guanidine which as a non-toxic polymer makes the catalyst more biodegradable and environmentally friendly. The synthesized catalyst can also be easily and rapidly separated from the reaction mixture by centrifugation. It can be recycled and recovered six times without significant loss in the catalytic activity. Also, this reaction also has a high atomic economy compared to other reactions.

**Table 4:** Comparison study on the catalytic efficiency of different surface modification

Catalyst	Yield (%)	Time (h)
Mesoporous PGTSA/Ni	93	24
Fe_3_O_4_/PGTSA/Ni	80	24
PGTSA/Ni	71	24

**Fig. 11:**
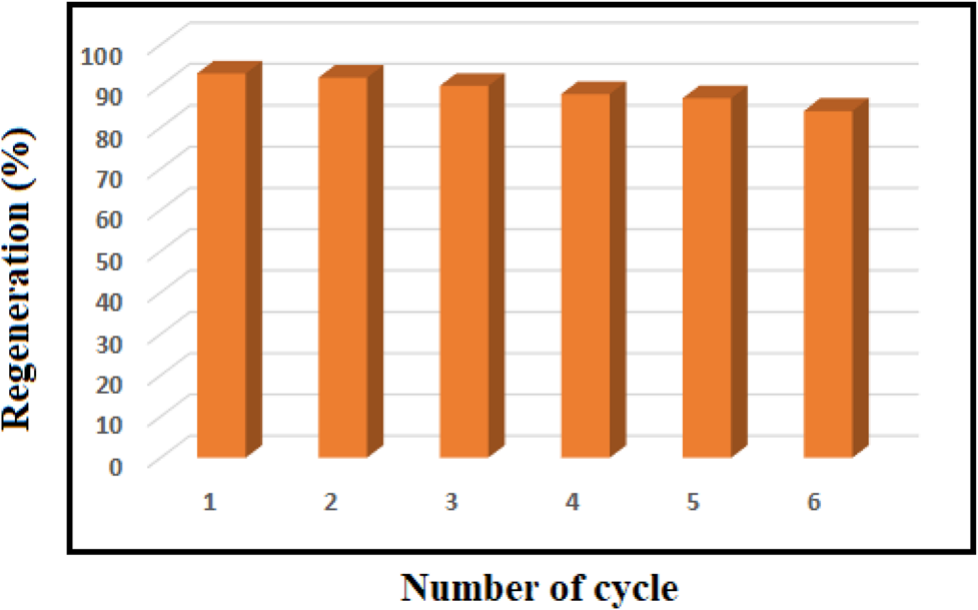
Recovery of nanocomposite in 6 runs for the model reaction.

The recyclability of mesoporous PGTSA/Ni was investigated toward synthesizing imines under optimized reaction conditions. Following completing the reaction, we segregated the catalyst from the reaction system through centrifugation, washed it several times with water and ethanol, and dried it in a vacuum drying oven at 80 °C for 4 h (Fig. 11).

## 4. Conclusions

We synthesized porous PGTSA using Fe_3_O_4_ MNPs as a hard template, followed by immobilization of Ni Cl_2_. The obtained solid was a combination of acidity of –NH groups, basicity, hydrogen bonding, and Lewis acidity of Ni^2+^ metal. This product was used as a heterogeneous active catalyst for acceptorless heterocoupling of amines into imines. The results showed the higher catalytic activity of PGTSA/Ni than the NiCl_2_ and CoCl_2_. The observed high catalytic activity might be attributed to the synergistic interaction between NiCl_2_ and porous PGTSA. Large surface area and high porosity are other important factors in catalytic activity. In addition to the high porosity and surface area, the high performance appears to arise from immobilization of the Ni complex of the open framework of porous PGTSA. Nickel species play a more important role in catalytic activity. Morever, PGTSA/Ni was found as a multifunctional catalyst with robustness and stability under reaction conditions. The product can be reused up to six times without any loss in its catalytic activity and structural integrity. The other benefits of the proposed mesoporous catalyst include high yield, facile separation of the catalyst from the mixture by centrifugation, great TOF, and a clean reaction profile. Overall, this research portrays a bright future for using porous polysulfonamides and their functionalized analogs as multifunctional catalysts.

## Conflicts of interest

There are no conflicts to declare.

## Supplementary Material
